# Supplementary material for: Unveiling complex patterns: An information-theoretic approach to high-order behaviors in microarray data
Source: PLoS One. 2025 Nov 13;20(11):e0336379. doi: 10.1371/journal.pone.0336379 (PMC12614557; doi:10.1371/journal.pone.0336379)
Supplement: S1 Appendix — (PDF) [file pone.0336379.s001.pdf]

S1 Appendix: HCC

Comm 29

List of Differentially expressed genes

- Entire Dataset, 7 DGs: TBXA2R, INMT, SULF2, OLFML3, ANGPTL1, SRPX, PDGFRA.
- Synergy Cluster 1, 8 DGs: TBXA2R, SULF2, INMT, TCF21, OLFML3, RBP1, PDGFRA, TACSTD2.
- Synergy Cluster 2, 7 DGs: OLFML3, INMT, SULF2, TBXA2R, SRPX, ANGPTL1, PDGFRA.
- MI Cluster 1, 8 DGs: SULF2, INMT, ANGPTL1, PDGFRA, TBXA2R, OLFML3, SRPX, TACSTD2.
- MI Cluster 3, 5 DGs: OLFML3, TBXA2R, ANGPTL1, SULF2, INMT.

As we can see, we gain three new DGs from Synergy cluster 1 and a new one from MI cluster 1. Reported below are the empirical p-values obtained by comparing observed gene-level statistics in these clusters to null distributions generated from 1,000 random subject subsets of identical size and class composition.

Gene	Emp_Pval
TBXA2R	0.24
SULF2	0.99
INMT	0.07
**TCF21**	0.02
OLFML3	0.75
**RBP1**	0.12
PDGFRA	0.42
**TACSTD2**	0.96

(a) Empirical p-value analysis for synergy cluster 1.

Gene	Emp_Pval
SULF2	0.60
INMT	0.35
ANGPTL1	0.99
PDGFRA	0.74
TBXA2R	0.90
OLFML3	0.99
SRPX	0.31
**TACSTD2**	0.15

(b) Empirical p-value analysis for MI cluster 1.

Statistical reinforcement analysis

For each community, we evaluated the accuracy value on the independent dataset and enriched biological functions on random boostrap samples. As the dimensionality of the subcommunity, we chose the number of genes included in the synergy cluster that is biologically interesting. For the Comm 29, the relevant Synergy cluster is the second one composed by 19 genes. The mean accuracy evaluated on boostrap sample is equal to $(76.61 \pm 5.92)\%$, S1 Figure. As we can see, the result obtained by the Synergy cluster is within the confidence interval.

Regarding enriched functions, the 80% of the functions found statistically significant are common to those given by the whole community and MI subcommunities. In contrast, there is no overlap with the functions found by the Synergy subcommunities. Listed below are the remaining functions:

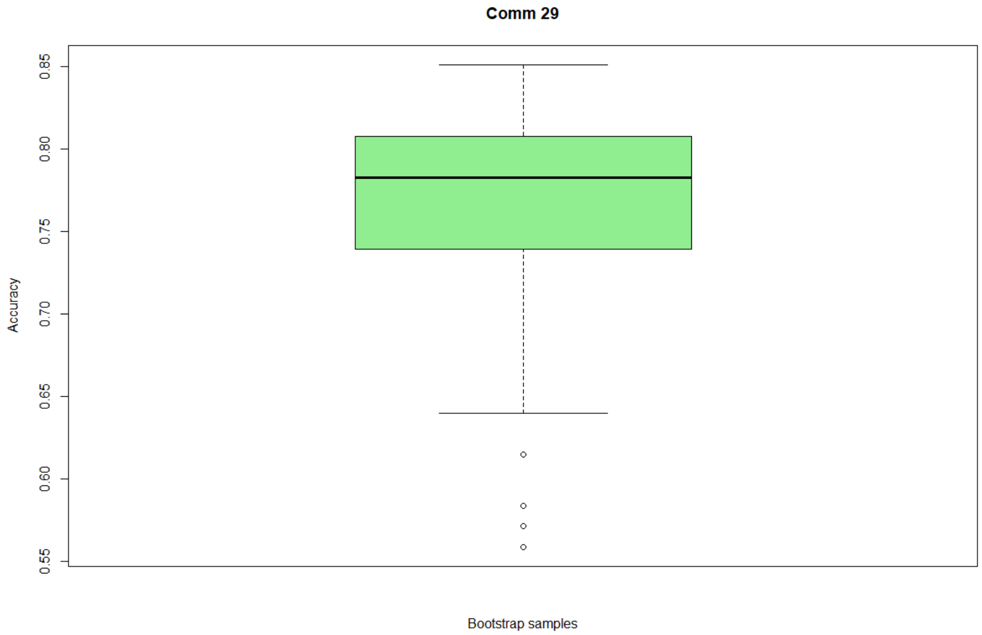

S1 Figure. Boxplot of the Accuracy of the Comm 29 evaluated on the independent set by random boostrap samples.

Gene Set Name	Genes in Overlap	Overlap Ratio	FDR q value
GOBP_REGULATION_OF_MORPHOGENESIS_OF_A_BRANCHING_STRUCTURE	3	0.0566	8.2E-3
GOBP_PROSTATE_GLANDULAR_ACINUS_MORPHOGENESIS	2	0.3333	9.49E-3
GOBP_CELL_PROJECTION_ORGANIZATION	7	0.0043	1.04E-2
HP_ABNORMAL_TRACHEOBRONCHIAL_MORPHOLOGY	5	0.0132	1.95E-3
HP_EXCESSIVE_WRINKLED_SKIN	3	0.0769	2.01E-3
GOBP_POSITIVE_REGULATION_OF_DEVELOPMENTAL_PROCESS	7	0.0050	3.31E-3
SWEET_LUNG_CANCER_KRAS_DN	6	0.0142	2.62E-4
PLASARI_NFIC_TARGETS_BASAL_DN	3	0.1364	5.67E-4
PLASARI_TGFB1_SIGNALING_VIA_NFIC_10HR_DN	3	0.0909	1.2E-3
PLASARI_TGFB1_SIGNALING_VIA_NFIC_1HR_UP	3	0.0909	1.2E-3
HP_FUNCTIONAL_ABNORMALITY_OF_THE_MIDDLE_EAR	5	0.0134	1.4E-3
HP_CONICAL_INCISOR	3	0.0789	1.48E-3
GOBP_TISSUE_MIGRATION	5	0.0130	2.46E-3
GOBP_AMEBOIDAL_TYPE_CELL_MIGRATION	5	0.0100	6.68E-3
HP_WHEEZING	3	0.0469	7.22E-3
GOBP_REGULATION_OF_CELL_POPULATION_PROLIFERATION	10	0.0056	2.72E-5
GOBP_ODONTOGENESIS	4	0.0299	2.13E-3
HALLMARK_APICAL_SURFACE	3	0.0682	3.87E-3
HP_MALABSORPTION	4	0.0165	1.09E-2

List of non common Enrichment Functions for the Bootstrap Clusters of Community 29.

Comm 32

List of Differentially expressed genes

- Entire Dataset, 35 DGs: CPB2, MAOB, HAO1, DEPDC7, HSD17B4, HNF4A, ALDH2, EPHX1, LPA /// PLG, SALL1, BDH1, HFE2, DMGDH, LOC102723493, AR, LOC101929880 /// QPRT, ALDH6A1, CES2, GPT2, HPD, PRAP1, SLC27A5, ALAS1, ACSM5, ABHD14B, ADH4, HSD11B1, APOA5, CYP2C9, CYP8B1, LOC149703, GLYAT, LOC101928230, RTP3, SEC14L2.
- Synergy Cluster 1, 35 DGs: CPB2, MAOB, HAO1, DEPDC7, HSD17B4, HNF4A, ALDH2, EPHX1, LPA /// PLG, SALL1, BDH1, HFE2, DMGDH, LOC102723493, AR, LOC101929880 /// QPRT, ALDH6A1, CES2, GPT2, HPD,

PRAP1, SLC27A5, ALAS1, ACSM5, ABHD14B, ADH4, HSD11B1, APOA5, CYP2C9, CYP8B1, LOC149703, GLYAT, LOC101928230, RTP3, SEC14L2.

- Synergy Cluster 2, 36 DGs: CPB2, MAOB, HAO1, DEPDC7, HSD17B4, HNF4A, ALDH2, EPHX1, LPA /// PLG, SALL1, BDH1, HFE2, DMGDH, LOC102723493, AR, LOC101929880 /// QPRT, ALDH6A1, CES2, GPT2, HPD, PRAP1, SLC27A5, ALAS1, ACSM5, ABHD14B, ADH4, HSD11B1, APOA5, CYP2C9, CYP8B1, LOC149703, GLYAT, LOC101928230, RTP3, SEC14L2, NAGS.
- Synergy Cluster 3, 36 DGs: CPB2, MAOB, HAO1, DEPDC7, HSD17B4, HNF4A, ALDH2, EPHX1, LPA /// PLG, SALL1, BDH1, HFE2, DMGDH, LOC102723493, AR, LOC101929880 /// QPRT, ALDH6A1, CES2, GPT2, HPD, PRAP1, SLC27A5, ALAS1, ACSM5, ABHD14B, ADH4, HSD11B1, APOA5, CYP2C9, CYP8B1, LOC149703, GLYAT, LOC101928230, RTP3, SEC14L2, NAGS.
- MI Cluster 1, 36 DGs: CPB2, MAOB, HAO1, DEPDC7, HSD17B4, HNF4A, ALDH2, EPHX1, LPA /// PLG, SALL1, BDH1, HFE2, DMGDH, LOC102723493, AR, LOC101929880 /// QPRT, ALDH6A1, CES2, GPT2, HPD, PRAP1, SLC27A5, ALAS1, ACSM5, ABHD14B, ADH4, HSD11B1, APOA5, CYP2C9, CYP8B1, LOC149703, GLYAT, LOC101928230, RTP3, SEC14L2, CYP7A1.

As we can see, we gain a new DGs from Synergy cluster 2 and 3, and MI cluster 1. Reported below are the empirical p-values obtained by comparing observed gene-level statistics in these clusters to null distributions generated from 1,000 random subject subsets of identical size and class composition. Since we obtain the same gene from synergy clusters 2 and 3, we report the table with empirical p-values only for cluster 2 for simplicity.

Statistical reinforcement analysis

For Community 32, both synergy clusters are relevant and have similar dimensionality going to split the starting cluster in half. Here the peculiarity of synergy was that it was able to find structures within a community of low dimensionality. Instead MI in fact failed to divide the community by returning a cluster comparable with the whole community. Thus by randomly sampling obviously we get biologically enriched features that also overlap with synergistic clusters.

The mean accuracy calculated from the bootstrap samples is $(75.22 \pm 6.92)\%$ (refer to Figure S2). Notably, the results achieved by the synergy cluster fall within the confidence interval.

**Gene**	**Emp_Pval**
CPB2	0.70
HAO1	0.28
EPHX1	0.96
HSD17B4	0.57
HNF4A	0.63
DEPDC7	0.96
LPA /// PLG	0.74
HFE2	0.59
BDH1	0.89
SALL1	0.34
ALDH2	0.87
DMGDH	0.97
AR	0.73
MAOB	0.85
CES2	0.93
LOC101929880 /// QPRT	0.41
GPT2	0.46
PRAP1	0.26
LOC102723493	0.81
ALAS1	0.91
SLC27A5	0.91
ACSM5	0.93
ALDH6A1	0.78
ABHD14B	0.70
**NAGS**	0.91
CYP2C9	0.93
RTP3	0.85
HPD	0.36
APOA5	0.58
HSD11B1	0.80
ADH4	0.92
CYP8B1	0.85
LOC149703	0.70
LOC101928230	0.97
GLYAT	0.86
SEC14L2	0.76

(a) Empirical p-value analysis for synergy cluster 2.

**Gene**	**Emp_Pval**
CPB2	0.39
LPA /// PLG	0.95
MAOB	0.45
HNF4A	0.25
HAO1	0.94
DEPDC7	0.14
SALL1	0.40
BDH1	0.37
EPHX1	0.72
ALDH2	0.99
HSD17B4	0.07
DMGDH	0.02
HFE2	0.82
HPD	0.26
AR	0.16
LOC102723493	0.62
ALDH6A1	0.92
CES2	0.74
ACSM5	0.87
ADH4	0.60
HSD11B1	0.77
PRAP1	0.19
GPT2	0.56
SLC27A5	0.44
LOC101929880 /// QPRT	0.08
APOA5	0.88
ALAS1	0.90
ABHD14B	0.18
CYP2C9	0.34
GLYAT	0.22
CYP8B1	0.77
LOC149703	0.46
RTP3	0.01
LOC101928230	0.32
SEC14L2	0.09
**CYP7A1**	0.98

(b) Empirical p-value analysis for MI cluster 1.

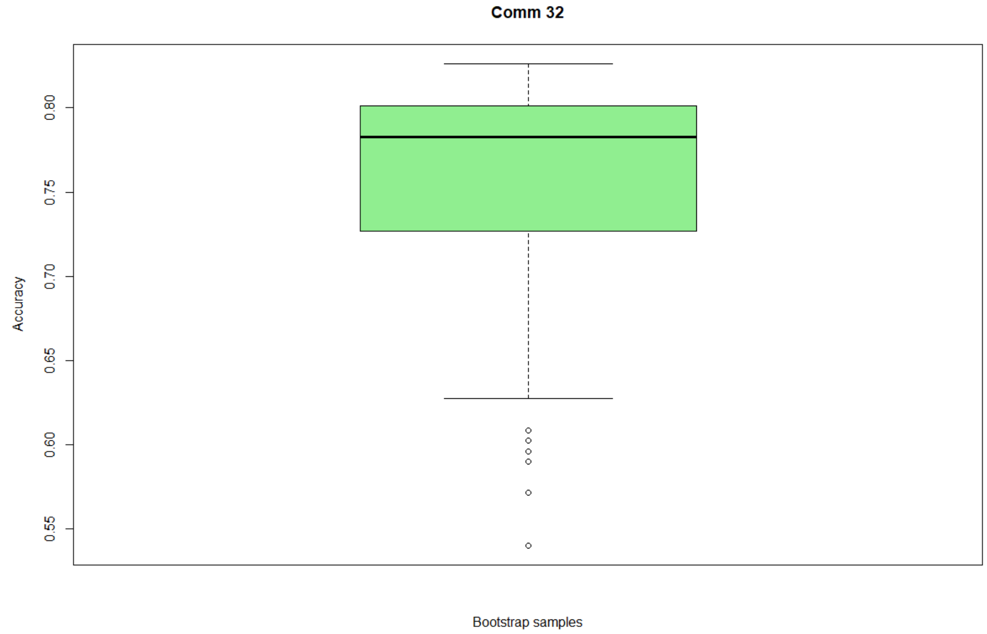

S2 Figure. Boxplot of the Accuracy of the Comm 32 evaluated on the independent set by random boostrap samples.
