# Supplementary material for: Unveiling complex patterns: An information-theoretic approach to high-order behaviors in microarray data
Source: PLoS One. 2025 Nov 13;20(11):e0336379. doi: 10.1371/journal.pone.0336379 (PMC12614557; doi:10.1371/journal.pone.0336379)
Supplement: S2 Appendix — (PDF) [file pone.0336379.s002.pdf]

S2 Appendix: ASD

Comm 50

List of Differentially expressed genes

- Entire Dataset, 6 DGs: FOXN1, PSKH1, FOXE3, CRB3, SPERT, EPHB4;
- Synergy Cluster 1, 8 DGs: SLC26A11, PPP1R9B, GPR150, PBX1, RXRG, LOC286526, ZNF706, RUFY3;
- Synergy Cluster 2, 3 DGs: PSKH1, SPERT, EPHB4;
- MI Cluster 1, 7 DGs: MGC40222, FOXN1, WAC, PSKH1, FOXE3, SPERT, EPHB4;
- MI Cluster 2, 2 DGs: PRKCBP1, LOC286526.

As we can see from the list above, we gain eight new DGs from Synergy cluster 1 and four new ones from MI cluster 1 and 2. Reported below are the empirical p-values obtained by comparing observed gene-level statistics in these clusters to null distributions generated from 1,000 random subject subsets of identical size and class composition.

Gene	Emp_Pval
**SLC26A11**	0.14
**PPP1R9B**	0.025
**GPR150**	0.99
**PBX1**	1
**RXRG**	0.99
**LOC286526**	0.01
**ZNF706**	0.01
**RUFY3**	0.20

(a) Empirical p-value analysis for synergy cluster 1.

Gene	Emp_Pval
**MGC40222**	0.84
FOXN1	0.91
**WAC**	0.094
PSKH1	0.13
FOXE3	0.016
SPERT	0.89
EPHB4	0.95

(b) Empirical p-value analysis for MI cluster 1.

Gene	Emp_Pval
**PRKCBP1**	0.82
**LOC286526**	0.87

(c) Empirical p-value analysis for MI cluster 2.

Statistical reinforcement analysis

The same reinforcement analysis done for HCC we repeated for ASD. For community 50 the synergy cluster from which we were able to extract enriched biological functions is the second one consisting of 9 genes. By creating random boostrap samples of this dimensionality we obtain no statistically significant function confirming the non-randomness of the synergy cluster.

Comm 78

List of Differentially expressed genes

- Entire Dataset, 15 DGs: CDK5RAP3, NBR2, LOC647881, LOC399900, CRYAA, MGAM, TMPRSS3, MME, GPR62, POP1, C18ORF24, KIF4A, HBD, FAM10A4, LOC285453;
- Synergy Cluster 1, 12 DGs: CDK5RAP3, NBR2, LOC399900, CRYAA, TMPRSS3, POP1, C18ORF24, CORO7, KIF4A, HBD, FAM10A4, LOC285453;

- Synergy Cluster 2, 2 DGs: LOC399900, EVX2;
- MI Cluster 1, 12 DGs: CDK5RAP3, NBR2, LOC647881, OR52N2, MOGAT2, LOC399900, MGAM, MME, GP1BA, IL15RA, KIF4A, FAM10A4.

As we can see from the list above, we gain two new DGs from Synergy cluster 1 and 2 and four new ones from MI cluster 1. Reported below are the empirical p-values obtained by comparing observed gene-level statistics in these clusters to null distributions generated from 1,000 random subject subsets of identical size and class composition.

**Gene**	**Emp_Pval**
CDK5RAP3	0.034
NBR2	0.15
LOC399900	0.26
CRYAA	0.35
TMPRSS3	0.34
POP1	0.22
C18ORF24	0.52
**CORO7**	0.48
KIF4A	0.21
HBD	0.12
FAM10A4	0.32
LOC285453	0.21

(a) Empirical p-value analysis for synergy cluster 1.

**Gene**	**Emp_Pval**
LOC399900	0.49
**EVX2**	0.98

(b) Empirical p-value analysis for synergy cluster 2.

**Gene**	**Emp_Pval**
CDK5RAP3	0.82
NBR2	0.60
LOC647881	0.81
**OR52N2**	0.01
**MOGAT2**	0.01
LOC399900	0.04
MGAM	0.01
MME	0.30
**GP1BA**	0.20
**IL15RA**	0.92
KIF4A	0.01
FAM10A4	0.01

(c) Empirical p-value analysis for MI cluster 1.

Statistical reinforcement analysis

For Community 78, the synergy cluster used to extract enriched biological functions is the third one, comprising 17 genes. When generating random bootstrap samples of this dimensionality, no statistically significant functions are obtained, further confirming the non-randomness of the synergy cluster.
