# Supplementary material for: Unveiling complex patterns: An information-theoretic approach to high-order behaviors in microarray data
Source: PLoS One. 2025 Nov 13;20(11):e0336379. doi: 10.1371/journal.pone.0336379 (PMC12614557; doi:10.1371/journal.pone.0336379)
Supplement: S1 Table — (PDF) [file pone.0336379.s003.pdf]

Gene Set Name	Genes in Overlap	Overlap Ratio	FDR q value
GOBP_SODIUM_ION_TRANSPORT	5	0.0202	2.9E-4
ROY_WOUND_BLOOD_VESSEL_DN	3	0.1304	8.42E-4
BOQUEST_STEM_CELL_UP	5	0.0191	1.88E-3
GOBP_SODIUM_ION_TRANSMEMBRANE_TRANSPORT	4	0.0222	1.99E-3
GOBP_EPITHELIAL_CELL_PROLIFERATION	5	0.0102	3.23E-3
BRUINS_UVC_RESPONSE_VIA_TP53_GROUP_B	6	0.0108	3.86E-3
GOBP_NEGATIVE_REGULATION_OF_SERINE_TYPE_ENDOPEPTIDASE_ACTIVITY	2	0.4000	4.43E-3
BERTUCCI_INVASIVE_CARCINOMA_DUCTAL_VS_LOBULAR_DN	3	0.0652	4.49E-3
GOBP_NEGATIVE_REGULATION_OF_SERINE_TYPE_PEPTIDASE_ACTIVITY	2	0.3333	5.44E-3
GOMF_CALCIUM_DEPENDENT_PHOSPHOLIPID_BINDING	3	0.0536	6.88E-3
BENPORATH_EED_TARGETS	7	0.0066	8.23E-3
GOBP_REGULATION_OF_SERINE_TYPE_PEPTIDASE_ACTIVITY	2	0.2500	8.23E-3
GOBP_REGULATION_OF_TRANSPORT	7	0.0039	8.51E-3
GOBP_REGULATION_OF_SODIUM_ION_TRANSPORT	3	0.0353	8.51E-3
GOBP_BLOOD_VESSEL_MORPHOGENESIS	6	0.0085	8.65E-3
GOBP_GLOMERULUS_MORPHOGENESIS	2	0.2222	9.32E-3
GOBP_METANEPHRIC_GLOMERULUS_VASCULATURE_DEVELOPMENT	2	0.2222	9.32E-3
RIGGI_EWING_SARCOMA_PROGENITOR_UP	5	0.0114	9.73E-3
MCBRYAN_PUBERTAL_BREAST_3_4WK_UP	4	0.0187	9.73E-3
GOBP_REGULATION_OF_PHOSPHOLIPASE_A2_ACTIVITY	2	0.2000	1.02E-2
GOCC_CILIUM	6	0.0079	1.02E-2
GOBP_EPITHELIAL_FLUID_TRANSPORT	2	0.1818	1.03E-2
CARRILLOREIXACH_HEPATOBLASTOMA_VS_NORMAL_DN	6	0.0048	1.03E-2
GOBP_MONOATOMIC_ION_TRANSPORT	6	0.0047	1.03E-2
REACTOME_TRANSPORT_OF_SMALL_MOLECULES	5	0.0069	1.03E-2
GOBP_POSITIVE_REGULATION_OF_CELL_POPULATION_PROLIFERATION	6	0.0047	1.05E-2
KANG_AR_TARGETS_UP	2	0.1176	1.73E-2
GOBP_MESODERM_DEVELOPMENT	3	0.0221	1.81E-2
GOBP_EPITHELIAL_TUBE_FORMATION	3	0.0219	1.81E-2
GOMF_CHANNEL_REGULATOR_ACTIVITY	3	0.0214	1.84E-2
DE_YY1_TARGETS_UP	2	0.1000	1.94E-2
GOBP_POSITIVE_REGULATION_OF_TRANSPORT	5	0.0056	1.94E-2
GOBP_TUBE_FORMATION	3	0.0199	2.06E-2
GOBP_MORPHOGENESIS_OF_EMBRYONIC_EPITHELIUM	3	0.0197	2.06E-2
CHARAFE_BREAST_CANCER_LUMINAL_VS_MESENCHYMAL_UP	4	0.0088	2.06E-2
GOBP_LABYRINTHINE_LAYER_MORPHOGENESIS	2	0.0909	2.06E-2
GOBP_MORPHOGENESIS_OF_AN_EPITHELIAL_FOLD	2	0.0833	2.34E-2
GOBP_NEGATIVE_REGULATION_OF_KERATINOCYTE_PROLIFERATION	2	0.0833	2.34E-2
BENPORATH_NANOG_TARGETS	5	0.0051	2.5E-2
GOBP_INORGANIC_ANION_TRANSPORT	3	0.0174	2.5E-2
GOBP_BIOMINERAL_TISSUE_DEVELOPMENT	3	0.0172	2.53E-2
REACTOME_DISORDERS_OF_TRANSMEMBRANE_TRANSPORTERS	3	0.0169	2.58E-2
GOBP_EMBRYONIC_PLACENTA_MORPHOGENESIS	2	0.0741	2.58E-2
GOBP_MORPHOGENESIS_OF_AN_EPITHELIUM	4	0.0077	2.73E-2
GOBP_MONOATOMIC_ION_TRANSMEMBRANE_TRANSPORT	5	0.0048	2.73E-2
GOBP_SKELETAL_SYSTEM_DEVELOPMENT	4	0.0076	2.73E-2
GOBP_MONOATOMIC_CATION_TRANSPORT	5	0.0047	2.73E-2

S 1. List of Enrichment Functions for the MI Clusters of Community 29.
