# Supplementary material for: Unveiling complex patterns: An information-theoretic approach to high-order behaviors in microarray data
Source: PLoS One. 2025 Nov 13;20(11):e0336379. doi: 10.1371/journal.pone.0336379 (PMC12614557; doi:10.1371/journal.pone.0336379)
Supplement: S2 Table — (PDF) [file pone.0336379.s004.pdf]

Gene Set Name	Genes in Overlap	Overlap Ratio	FDR q value
GOBP_REGULATION_OF_LIPASE_ACTIVITY	4	0.0506	4.15E-4
OHM_METHYLATED_IN_ADULT_CANCERS	3	0.1111	7.69E-4
GOBP_REPRODUCTION	9	0.0059	1.18E-3
GOBP_WATER_TRANSPORT	3	0.1000	1.29E-3
GOBP_EMBRYONIC_DIGESTIVE_TRACT_DEVELOPMENT	3	0.0909	1.52E-3
GOBP_FLUID_TRANSPORT	3	0.0769	2.1E-3
GOBP_SENSORY_ORGAN_DEVELOPMENT	6	0.0102	2.46E-3
GOBP_EXTRACELLULAR_MATRIX_ASSEMBLY	3	0.0652	2.79E-3
GOCC_CELL_PROJECTION_MEMBRANE	5	0.0142	3.01E-3
GOBP_LUNG_ALVEOLUS_DEVELOPMENT	3	0.0612	3.13E-3
HOQUE_METHYLATED_IN_CANCER	3	0.0536	4.52E-3
MEISSNER_BRAIN_HCP_WITH_H3K4ME3_AND_H3K27ME3	7	0.0065	4.77E-3
CHICAS_RB1_TARGETS_GROWING	4	0.0165	6.46E-3
OHM_EMBRYONIC_CARCINOMA_DN	2	0.2500	7.88E-3
ROSS_AML_WITH_AML1_ETO_FUSION	3	0.0380	7.88E-3
SCHAEFFER_PROSTATE_DEVELOPMENT_AND_CANCER_BOX5_UP	2	0.1818	1.14E-2
NABA_SECRETED_FACTORS	4	0.0117	1.5E-2
GOBP_REGULATION_OF_VASCULATURE_DEVELOPMENT	4	0.0112	1.64E-2
GOBP_REGULATION_OF_ANATOMICAL_STRUCTURE_MORPHOGENESIS	5	0.0053	3.34E-2
CHARAFE_BREAST_CANCER_LUMINAL_VS_MESENCHYMAL_DN	4	0.0086	3.35E-2
STEGER_ADIPOGENESIS_DN	2	0.0800	3.67E-2
KEGG_MEDICUS_REFERENCE_WNT_SIGNALING_MODULATION_WNT_INHIBITOR	2	0.0714	4.21E-2
LOPES_METHYLATED_IN_COLON_CANCER_UP	2	0.0714	4.21E-2
GOBP_POSITIVE_REGULATION_OF_VASCULATURE_DEVELOPMENT	3	0.0162	4.21E-2
GOMF_WNT_PROTEIN_BINDING	2	0.0625	4.98E-2
GRUETZMANN_PANCREATIC_CANCER_DN	3	0.0150	4.98E-2

S 2. List of Enrichment Functions for the Synergy Clusters of Community 29.
