# Supplementary material for: Unveiling complex patterns: An information-theoretic approach to high-order behaviors in microarray data
Source: PLoS One. 2025 Nov 13;20(11):e0336379. doi: 10.1371/journal.pone.0336379 (PMC12614557; doi:10.1371/journal.pone.0336379)
Supplement: S3 Table — (PDF) [file pone.0336379.s005.pdf]

Gene Set Name	Genes in Overlap	Overlap Ratio	FDR q value
CAIRO_HEPATOBLASTOMA_DN	5	0.0187	1.13E-4
KEGG_GLYCINE_SERINE_AND_THREONINE_METABOLISM	3	0.0968	2.53E-4
GOCC_MITOCHONDRION	8	0.0048	3.33E-4
REACTOME_METABOLISM_OF_AMINO_ACIDS_AND_DERIVATIVES	5	0.0134	4.33E-4
DESERT_PERIVENOUS_HEPATOCELLULAR_CARCINOMA_SUBCLASS_UP	4	0.0258	4.33E-4
REACTOME_METABOLISM_OF_STEROIDS	4	0.0258	4.33E-4
GOMF_OXIDOREDUCTASE_ACTIVITY_ACTING_ON_THE_ALDEHYDE_OR _OXO_GROUP_OF_DONORS_NAD_OR_NADP_AS_ACCEPTOR	3	0.0769	5.93E-4
GOBP_FATTY_ACID_BIOSYNTHETIC_PROCESS	4	0.0238	6.67E-4
SU_LIVER	3	0.0517	1.22E-3
LEE_LIVER_CANCER_E2F1_DN	3	0.0469	1.58E-3
LEE_LIVER_CANCER_MYC_E2F1_DN	3	0.0469	1.58E-3
LEE_LIVER_CANCER_ACOX1_DN	3	0.0462	1.59E-3
KAPOSI_LIVER_CANCER_MET_DN	2	0.3333	1.86E-3
CHYLA_CBFA2T3_TARGETS_DN	4	0.0163	2.11E-3
KEGG_METABOLISM_OF_XENOBIOTICS_BY_CYTOCHROME_P450	3	0.0429	2.4E-3
ANDERSEN_LIVER_CANCER_KRT19_DN	3	0.0400	2.84E-3
GOBP_STEROL_HOMEOSTASIS	3	0.0297	6.7E-3
REACTOME_ETHANOL_OXIDATION	2	0.1667	6.75E-3
ACEVEDO_NORMAL_TISSUE_ADJACENT_TO_LIVER_TUMOR_DN	4	0.0113	7.65E-3
GOBP_UNSATURATED_FATTY_ACID_METABOLIC_PROCESS	3	0.0261	8.43E-3
GOBP_ALDEHYDE_CATABOLIC_PROCESS	2	0.1429	8.43E-3
GOBP_ALCOHOL_METABOLIC_PROCESS	4	0.0107	8.54E-3
WP_FATTY_ACID_OMEGA_OXIDATION	2	0.1333	9.16E-3
GOMF_ALDEHYDE_DEHYDROGENASE_NAD_P_PLUS_ACTIVITY	2	0.1176	1.12E-2
GOBP_ETHANOL_METABOLIC_PROCESS	2	0.0952	1.64E-2
GOBP_OLEFINIC_COMPOUND_METABOLIC_PROCESS	3	0.0185	1.86E-2
UEDA_PERIFERAL_CLOCK	3	0.0175	2.13E-2
GOBP_LIPID_HOMEOSTASIS	3	0.0171	2.17E-2
GOMF_STEROID_HYDROXYLASE_ACTIVITY	2	0.0769	2.17E-2
WP_PPAR_ALPHA_PATHWAY	2	0.0769	2.17E-2
GOBP_CHEMICAL_HOMEOSTASIS	5	0.0048	2.17E-2
WP_METAPATHWAY_BIOTRANSFORMATION_PHASE_I_AND_II	3	0.0164	2.32E-2
BERENJENO_ROCK_SIGNALING_NOT_VIA_RHOA_UP	2	0.0690	2.55E-2
GOBP_CELLULAR_RESPONSE_TO_XENOBIOTIC_STIMULUS	3	0.0156	2.56E-2
GOMF_TRANSITION_METAL_ION_BINDING	5	0.0045	2.61E-2

S 3. List of Enrichment Functions for the Synergy Clusters of Community 32.
