# Supplementary material for: Unveiling complex patterns: An information-theoretic approach to high-order behaviors in microarray data
Source: PLoS One. 2025 Nov 13;20(11):e0336379. doi: 10.1371/journal.pone.0336379 (PMC12614557; doi:10.1371/journal.pone.0336379)
Supplement: S4 Table — (PDF) [file pone.0336379.s006.pdf]

KEGG Pathway ID	KEGG Pathway Description	GeneRatio	FDR q value
*hsa*04927	Cortisol synthesis and secretion	2/4	3.00E-3
*hsa*04934	Cushing syndrome	2/4	8.48E-3
*hsa*05202	Transcriptional misregulation in cancer	2/4	8.72E-3
*hsa*04010	MAPK signaling pathway	2/4	1.56E-2

S 4. List of Enrichment Functions for the Synergy Clusters of Community 50.
