# Supplementary material for: Unveiling complex patterns: An information-theoretic approach to high-order behaviors in microarray data
Source: PLoS One. 2025 Nov 13;20(11):e0336379. doi: 10.1371/journal.pone.0336379 (PMC12614557; doi:10.1371/journal.pone.0336379)
Supplement: S5 Table — (PDF) [file pone.0336379.s007.pdf]

KEGG Pathway ID	KEGG Pathway Description	GeneRatio	FDR q value
*hsa*04975	Fat digestion and absorption	1/3	3.52E-2
*hsa*04978	Mineral absorption	1/3	3.52E-2
*hsa*00561	Glycerolipid metabolism	1/3	3.52E-2
*hsa*04137	Mitophagy - animal	1/3	3.52E-2
*hsa*04974	Protein digestion and absorption	1/3	3.52E-2

S 5. List of Enrichment Functions for the Synergy Clusters of Community 78.
