# Supplementary figures and images for: Unveiling complex patterns: An information-theoretic approach to high-order behaviors in microarray data
Source: PLoS One. 2025 Nov 13;20(11):e0336379. doi: 10.1371/journal.pone.0336379 (PMC12614557; doi:10.1371/journal.pone.0336379)

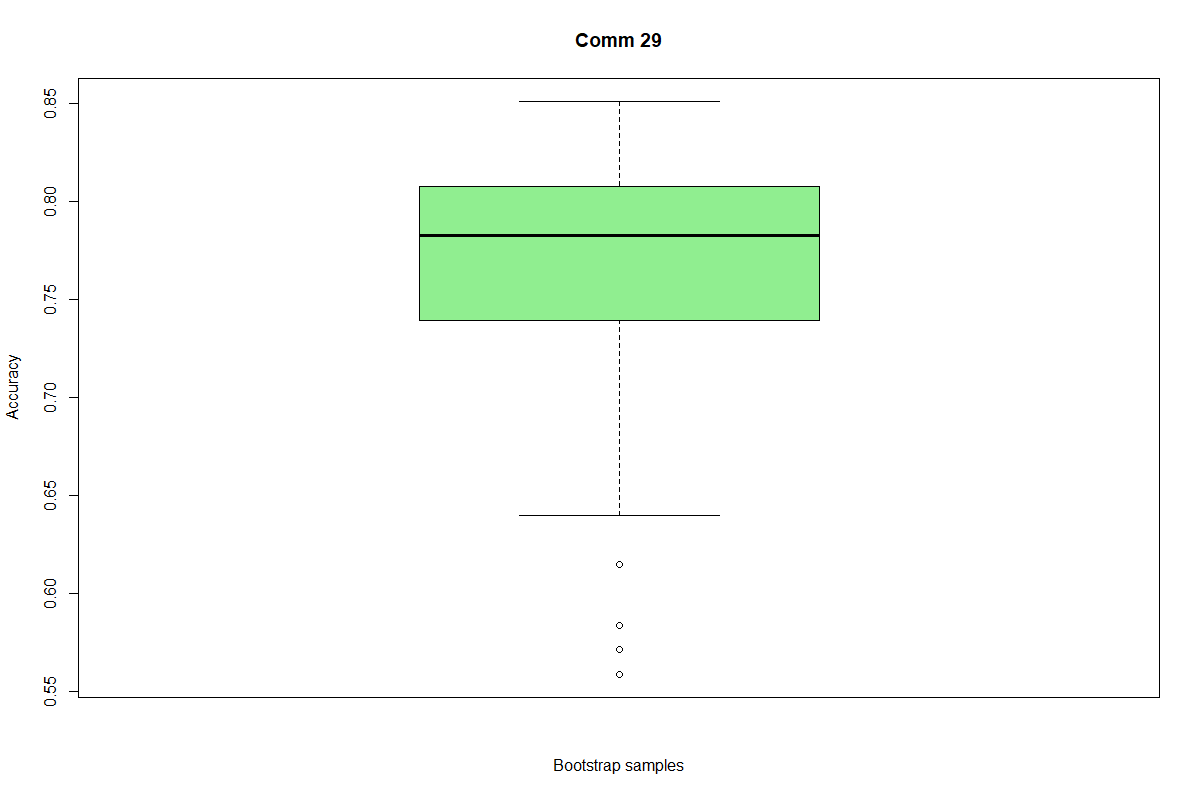

Supplement: S1 Fig — (PNG) [file pone.0336379.s008.png]

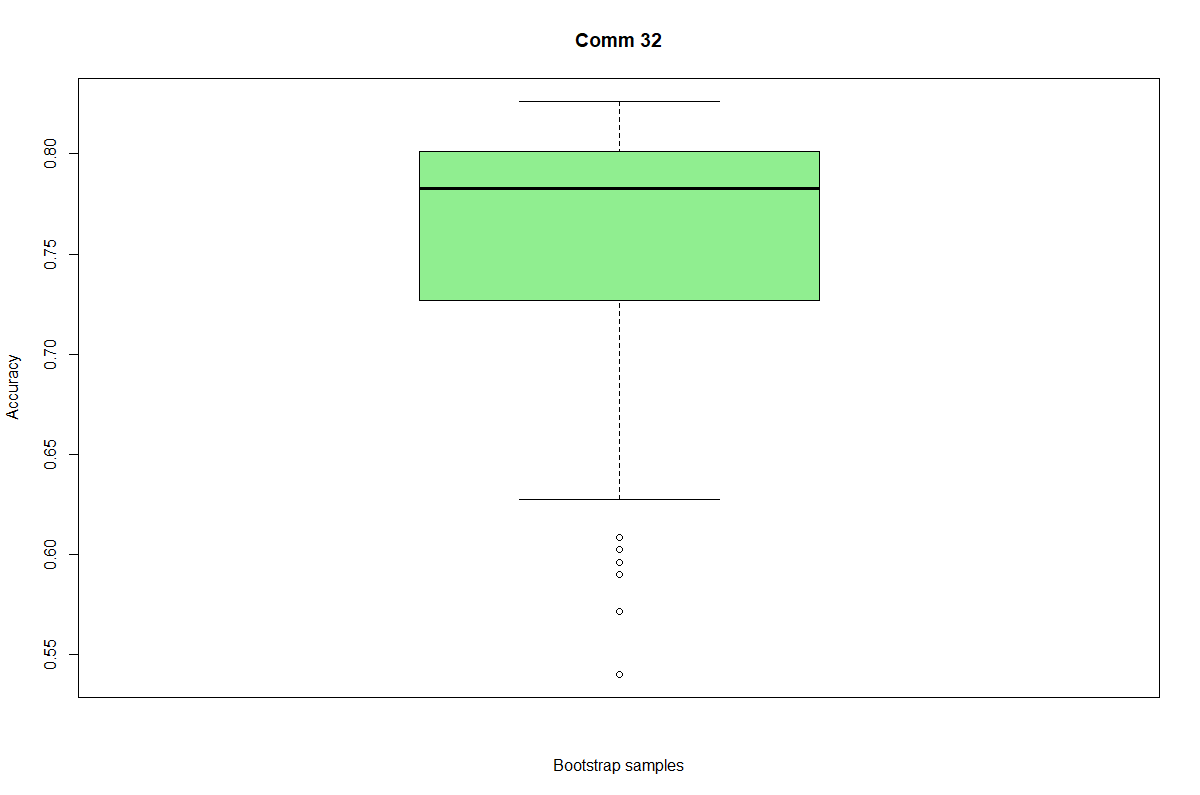

Supplement: S2 Fig — (PNG) [file pone.0336379.s009.png]
