# Supplementary material for: Unveiling complex patterns: An information-theoretic approach to high-order behaviors in microarray data
Source: PLoS One. 2025 Nov 13;20(11):e0336379. doi: 10.1371/journal.pone.0336379 (PMC12614557; doi:10.1371/journal.pone.0336379)
Supplement: Attachment — Submitted filename: point_to_point_answers_to_reviewers.pdf [file pone.0336379.s010.pdf]

PONE-D-24-38840
Unveiling Complex Patterns: An Information-Theoretic Approach to High-Order Behaviors in Microarray Data
PLOS ONE

The **weaknesses** of the manuscript include **clarity of language**, as some sentences are long and may cause ambiguity. For example, the phrase "*To assess the biological significance of our results, we used two biological tools: Differential Gene Expression (DGE) analysis and Pathways Analysis*" could be simplified for easier comprehension. The **excessive technical language** might make the manuscript less accessible to a broader audience. Regarding methodological innovation, while PID is an interesting approach, it is applied to previously published data, and the results **do not seem to introduce major advancements in the field**. The **limitations** of the PID method **are not sufficiently discussed**, especially in the context of extending its applications to other types of complex data. The analysis is restricted to only two clinical cases, and the **lack of clinical validation** or additional experiments reduces the practical applicability of the study. Additionally, the exploration of the data is sometimes superficial, and the **biological implications of the findings are not adequately developed**.

My recommendations include **rephrasing certain paragraphs** to improve clarity and avoiding excessive technical jargon. **The authors could discuss the biological implications of their findings in greater detail and should address the limitations of the PID method more explicitly in the discussion section**. **The conclusions could be more clearly defined**, **emphasizing how these methods could be extended to other diseases or datasets**.

Adding further analyses on additional datasets would provide broader validity and relevance to the results.
Interdisciplinary collaboration with clinicians or geneticists for the validation of the findings could add practical value and credibility to the study.

Please submit your revised manuscript by Feb 19 2025 11:59PM.

Journal Requirements:

When submitting your revision, we need you to address these additional requirements.

1. Please ensure that your manuscript meets **PLOS ONE's style requirements**, including those for **file naming**. The PLOS ONE style templates can be found at https://journals.plos.org/plosone/s/file?id=wjVg/PLOSOne_formatting_sample_main_body.pdf
and
https://journals.plos.org/plosone/s/file?id=ba62/PLOSOne_formatting_sample_title_authors_affiliations.pdf

Done

2. We noticed you have some **minor occurrence of overlapping text** with the following previous **publication**(s), which needs to be addressed:

-A statistical framework for neuroimaging data analysis based on mutual information estimated via a gaussian copula (doi: https://doi.org/10.1002/hbm.23471)

-DNA methylation fingerprint for the diagnosis and monitoring of hepatocellular carcinoma from tissue and liquid biopsies (doi: https://doi.org/10.1101/2021.06.01.21258144)

(among others)

In your **revision ensure you cite all your sources** (including your own works), and **quote or rephrase** any duplicated text outside the methods section. Further consideration is dependent on these concerns being addressed.

Done

3. We note that the **grant information** you provided in the 'Funding Information' and 'Financial Disclosure' sections **do not match**.

When you resubmit, please ensure that you provide the correct grant numbers for the awards you received for your study in the 'Funding Information' section.

Done

4. Please ensure that you **refer to Figure 5 in your text as**, if

accepted, production will need this reference to link the reader to the figure.
Done

5. We note you have **included a table** to which you **do not refer in the text** of your manuscript. **Please ensure that you refer to Table 1, 4 and 10 in your text**; if accepted, production will need this reference to link the reader to the Table.
Done

6. Please **include captions for your Supporting Information files at the end of your manuscript**, and **update any in-text citations to match accordingly**. Please see our Supporting Information guidelines for more information: http://journals.plos.org/plosone/s/supporting-information.

Done

Reviewers' comments:

Reviewer's Responses to Questions

Comments to the Author

1. Is the manuscript technically sound, and do the data support the conclusions?

The manuscript must describe a technically sound piece of scientific research with data that supports the conclusions. Experiments must have been conducted rigorously, with appropriate controls, replication, and sample sizes. The conclusions must be drawn appropriately based on the data presented.

Reviewer #1: Partly

Reviewer #2: No
----

2. Has the statistical analysis been performed appropriately and rigorously?

Reviewer #1: No

Reviewer #2: No
----

3. Have the authors made all data underlying the findings in their manuscript fully available?

The PLOS Data policy [3] requires authors to make all data underlying the findings described in their manuscript fully available without restriction, with rare exception (please refer to the Data Availability Statement in the manuscript PDF file). The data should be provided as part of the manuscript or its supporting information, or deposited to a public repository. For example, in addition to summary statistics, the data points behind means, medians and variance measures should be available. If there are restrictions on publicly sharing data--e.g. participant privacy or use of data from a third party--those must be specified.

Reviewer #1: Yes

Reviewer #2: Yes
----

4. Is the manuscript presented in an intelligible fashion and written in standard English?

PLOS ONE does not copyedit accepted manuscripts, so the language in submitted articles must be clear, correct, and unambiguous. Any typographical or grammatical errors should be corrected at revision, so please note any specific errors here.

Reviewer #1: Yes

Reviewer #2: Yes
----

5. Review Comments to the Author

Please use the space provided to explain your answers to the questions above. You may also include additional comments for the author, including concerns about dual publication, research ethics, or publication ethics. (Please upload your review as an attachment if it exceeds 20,000 characters)

Reviewer #1: General comment
In this paper, authors applied the partial information decomposition (PID) to two microarray gene expression datasets. In previous work, gene

communities were identified. The PID is used to explore possible hidden information not accessible using classical methods. Results are shown for hepatocellular carcinoma (HCC) and autism spectrum disorder (ASD) microarray data.

Specific comments

In the **material and methods section**, **authors should summarize their previous work results used in the present work**. The **content of the reference 32** may not be **available** to the reader and the **Figure 1 is not enough** to fully understand the previous work.

We thank the reviewer for his comment. In section experimental procedure we added further details about our 2 previous works.

In the **material and methods section**, a Venn diagram is used to explain the partial information decomposition for two sources X1 & X2, and a target Y. However, there is a lack of link between the sources X1 & X2, the target Y, and the gene communities. **It will be interesting to explain how the PID is applied to the gene communities to get the results presented.**

How the redundancy and the synergy have been computed for the gene communities?

The X and Y in the mutual information are random variables which distributions are used to get I(X;Y). **Are the sources X1 and X2 and the target Y random variables? How the mutual information is applied to clustering results and how the copula part is computed?**

We thank the reviewer for his comment. We added some sentences in section experimental procedure to better explain our PID approach.

Authors should explain why it is necessary to perform clustering and why using the PAM clustering method and not another. Otherwise, that are the links between clustering results and the PID.

We used PAM because it can be fed with dissimilarity matrices. Since with our approach we obtained square matrices whose element i,j is the redundancy or synergy between i and j, these matrices are dissimilarity matrices in all respects and therefore PAM seemed to be the most suitable algorithm.

Reviewer #2: In this manuscript, the authors present an innovative approach of combining Partial Information Decomposition (PID) with microarray data to demonstrate that it can pinpoint mechanisms of

complex disease. Their application, however, is limited to a set of genes that they have previously identified as capable of accurately distinguishing between disease and healthy states. This approach is highly limiting and is associated with two major problems.

1: The authors base their analysis on genes selected in their previous work (https://doi.org/10.3390/ijms242015286), making it challenging to critically assess the study without examining the gene selection process described in their prior research. According to their previous work, the gene communities identified are highly correlated. **Highly correlated gene sets inflate redundancy.** Thus, **it is not surprising that subsets of redundant genes that are previously known to separate disease and healthy states would perform well.** The claims that some of the synergy clusters outperform MI (Mutual Information) clusters and the whole gene set **is not substantiated by evidence**. **Performance metrics summarized in Table 3 are very close to each other and without confidence intervals, it is not possible to evaluate the performance of SI (Synergistic Information) cluster genes.**

The aim of our work is to start from gene communities already found in our previous analyses to perform well in discriminating between healthy and unhealthy subjects, and to verify the presence of informative subsamples within these communities. To this end, we considered a more classical approach using clusters based on mutual information and a "high order" approach using gene clusters from PID. Our results show that in general, only in the Synergy clusters, compared to the whole community benchmark, we found significantly enriched biological functions or in some cases even differentially expressed genes closely linked to the disease phenotype. These results are not found in the MI clusters. In addition, we checked the performances of the new gene sub-communities found with the PID approach in the classification of healthy and unhealthy subjects and found that these sub-communities matched and in some cases even exceeded the performances of the whole community, but with a lower number of genes.

The results shown in Table 3 refer to the independent test and not to the cross-validation procedure, so it was not possible to assign a confidence interval to the accuracy measures.

2: The authors aim to demonstrate that when PID is applied to microarray data, it can pinpoint mechanisms of complex disease. Nonetheless, the data do not fully support this conclusion. For example, the authors claim that synergy maximizes the variability of the samples and leads to detection of a greater number of disease relevant genes. However, small differences in the number of differentially expressed genes derived from within cluster comparisons can be due to chance. **The authors should combine bootstrapping and differential gene expression analysis to show that the small differences are not due to chance.**

We introduced a bootstrapping procedure to check the robustness of our results.
We have added some sentences to the Experimental procedure section to better explain our procedure.
The results obtained, which are presented in the Results section of the main text and in the supplementary materials, demonstrate the robustness of our results.

We report the results obtained below:
For HCC

- Community 29: The classification accuracy of the bootstrap subsets is statistically consistent with the results obtained with both MI and Syn. The enriched biological functions of the random subsamples overlap mostly (>90%) with the function of the whole dataset and the MI subsets. There is no overlap with the Synergy clusters.
- Comm 32: The classification accuracy of the bootstrap subsets is statistically consistent with that obtained with MI and Syn. The enriched biological functions found overlap with those of Synergy, but probably because the community is not large. Also note that MI did not succeed in splitting the community into sub-clusters.

For ASD:

- Community 50: Bootstrap subsamples do not provide enriched characteristics (as with the whole community and MI sub-communities).
- Community 78: Bootstrap samples do not provide enriched characteristics (as in the whole community and MI sub-communities).

To avoid preselection biases, **PID should be directly applied to all genes or to a biologically informed subsets** (e.g., gene ontology).

The PID method is very computationally intensive, so it was not possible to apply it to the original dataset, which included thousands of genes (analysed in the two earlier papers from which we start). We have indicated in a table the possible combinations (triplets) depending on the number of genes considered with the computation times. In figure below you can see that with a sample of 1000 genes the computational estimated time is about 300 years.
We used a server with 16 CPUs and 64 GBytes of RAM.
Instead, PID was applied directly to the most informative communities found in the 2 previous papers in order to limit the number of possible combinations and eliminate genes that were found to be not very informative in previous analyses.
We added some sentences in section discussion among the limitations of our work.

Genes	50	100	200	500	1000
Combinations	59k	485k	3.9M	62M	498M
Computation	few	few minutes	about 1h	about 7 days	estimated:

Time of PID	seconds				about 300 years

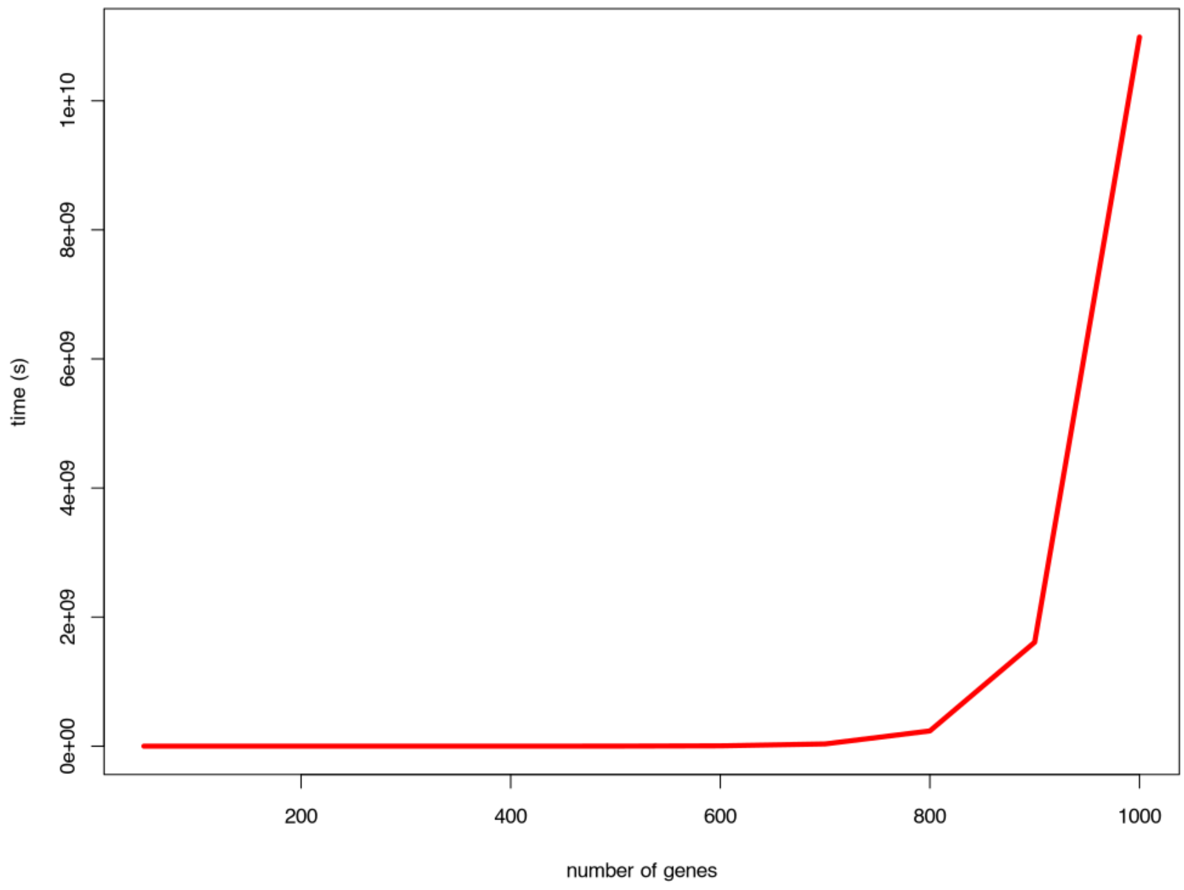

Benchmarking MI against SI utilizing high-throughput data has potential; however, in it is current form, I cannot recommend this manuscript for publication.

Minor Issues:

- The Material and Methods section should include more detail including a codebase to encourage reproducibility. Also, all datasets used in the paper should be explicitly indicated in the Materials and Methods section. For example, GSE54236 is only mentioned in the Results section.

We have included in the paper the information about all datasets used. We have uploaded all codes used at a public repository: https://github.com/alacalamita95/Plos_One_PID..

- The same statistical test for identifying differentially expressed genes should be used throughout the paper.

We used DGE with limma package for testing both HCC and ASD. For ASD, DGE did not provide statistically significant results so we tried to investigate further using KS-test which is a completely different statistical test from DGE.

.

- "Transmission" is misspelled in the abstract.

Done

----

6. PLOS authors have the option to publish the peer review history of their article (what does this mean? [4]). If published, this will include your full peer review and any attached files.

If you choose "no", your identity will remain anonymous but your review may still be made public.

Do you want your identity to be public for this peer review? For information about this choice, including consent withdrawal, please see our Privacy Policy [5].

Reviewer #1: Yes: Doulaye DEMBELE

Reviewer #2: No
