# Supplementary material for: Unveiling complex patterns: An information-theoretic approach to high-order behaviors in microarray data
Source: PLoS One. 2025 Nov 13;20(11):e0336379. doi: 10.1371/journal.pone.0336379 (PMC12614557; doi:10.1371/journal.pone.0336379)
Supplement: Attachment — Submitted filename: point_to_point_answers_to_reviewers_new.pdf [file pone.0336379.s011.pdf]

Dear Dr. Monaco,

Thank you for submitting your manuscript to PLOS ONE. After careful consideration, we feel that it has merit but does not fully meet PLOS ONE's publication criteria as it currently stands. Therefore, we invite you to submit a revised version of the manuscript that addresses the points raised during the review process.

Please submit your revised manuscript by Aug 27 2025 11:59PM. If you will need more time than this to complete your revisions, please reply to this message or contact the journal office at plosone@plos.org. When you're ready to submit your revision, log on to https://www.editorialmanager.com/pone/ and select the 'Submissions Needing Revision' folder to locate your manuscript file.

Additional Editor Comments:

Since one of reviewers was very nagative, please try to address all the concerns raised by reviewers as much as possible.

6. Review Comments to the Author

Reviewer #1: The contribution of this paper is the use of the PID to microarray gene expression analysis. However, the superiority of the PID approach is not shown over the methods used for gene set enrichment analysis.

We appreciate the reviewer highlighting this aspect. The advantage and novelty of applying Partial Information Decomposition (PID) is precisely its capability to unveil non-casual internal substructures within previously identified gene communities. These substructures, which emerge from higher-order interactions captured specifically by synergy (a multivariate measure), are not identified by classical bivariate metrics such as Mutual Information alone. As clearly reported in the "Results" and "Discussion" sections, applying PID allowed us to identify specific gene subsets within communities previously found to have no biologically enriched functions, uncovering significant biological associations specifically linked to the diseases analyzed (HCC and ASD).

Specifically, as we detailed in the manuscript:

- For the HCC use case (community 32), PID identified gene subclusters enriched with functions associated with pediatric and adult liver cancers, despite the original entire community showing no enrichment.
- For the ASD use case (community 78), PID unveiled gene subclusters significantly associated with gastrointestinal disorders commonly linked to ASD, whereas no biological enrichment was initially detected in the whole community.

Thus, the superiority of PID in our analysis lies precisely in its ability to detect biologically meaningful internal structures previously hidden when applying traditional methods alone. To further clarify this point in the manuscript, we added the following sentence at the end of the "Discussion" section:

"The superiority of PID in this study is highlighted by its unique ability to reveal internal structures within previously identified gene communities, which classical gene enrichment methods alone did not detect."

The calculation of the PID components from a gene community is **not explained in the text** and there is **no biological validation** of the findings.

We agree with the reviewer on the importance of clearly explaining the calculation procedure. We carefully described the procedure in the "Experimental procedure" subsection within the "Materials and methods" section, detailing the steps:

- Evaluating synergy for each possible triplet combination of nodes (samples or genes). This is done by solving the 4 equations of the PID Theory.
- Averaging these synergy values to generate a dissimilarity matrix.
- Employing silhouette analysis to determine the optimal number of clusters.
- Using Partitioning Around Medoids (PAM) to form the clusters based on the PID metrics.

To make this even clearer, we added the following clarifying sentence to the "Experimental procedure" subsection:

> "Specifically, PID components (synergy, redundancy, and unique information) were calculated for each possible triplet combination of nodes (samples or genes) within a community, thus capturing higher-order interactions that traditional pairwise methods do not account for."

We respectfully clarify that we have provided biological validation through Differential Gene Expression (DGE) analysis and enrichment analyses, described in the "Results" and further elaborated in the "Discussion" sections. This biological validation demonstrated how the PID-derived clusters unveiled gene subsets specifically enriched with known biological functions and genes directly linked to the diseases studied, providing robust biological insights:

- For HCC, we reported validated cancer-related genes (e.g., TCF21, RBP1) and enriched biological pathways (e.g., Wnt/β-catenin signaling).
- For ASD, we identified genes such as RXRG and PBX1 previously associated with neurodevelopmental disorders and enriched functions related to cortisol regulation and gastrointestinal disorders known to be associated with ASD.

To reinforce the clarity and emphasis of biological validation, we added the following sentence to the "Biological tool" subsection in the "Materials and methods" section:

> "The biological validation of PID-derived clusters was achieved through this combined approach of identifying differential gene expressions and subsequently assessing pathway enrichments, explicitly demonstrating the biological significance of higher-order interactions identified through PID."

The **silhouette** measure allows us to evaluate a clustering method result. Authors should explain how they use the silhouette for determining the optimal number of clusters.

We thank the reviewer for highlighting this important methodological detail. We used the standard approach to determine the optimal number of clusters, specifically by calculating the silhouette score for cluster numbers ranging from 2 to 10. The optimal number of clusters was chosen as the one that maximizes the silhouette score, indicating the best trade-off between cohesion and separation of clusters. We added this sentence in the the "Experimental procedure" subsection:

> "The silhouette coefficient was evaluated for clustering solutions ranging from 2 to 10 clusters, selecting the number that maximized the silhouette score."

The **differential gene expression** analysis is usually done at the genome level to select a subset of genes for functional analysis. LIMMA is not a biological function enrichment tool.

We agree with the reviewer and thank them for this clarification. In our manuscript, we indeed used LIMMA solely to identify differentially expressed genes (DGs) between clusters, not as a biological enrichment tool. After identifying these DGs, we subsequently performed gene set enrichment analysis using specific tools clearly mentioned in the manuscript (GSEA for HCC and KEGG via clusterProfiler for ASD) to assess the biological relevance of the PID-derived subclusters. To clarify this further detail, we entirely reviewed the "Biological tool" subsection.

We modified other sentences in the paper to clarify this aspect.

Reviewer #2: The authors identified informative sets of subsamples by applying partial information decomposition (PID) on gene communities that was previously shown to distinguish healthy and disease states. Here, they also showed that when the informative cluster space is repeatedly bootstrapped using the same number of genes as the synergy cluster, the **predictive performance** of these random samples is comparable to that of the synergy cluster. This suggests that a random subsample of a similarly sized gene space can **perform** equally well in classifying healthy and disease states. Thus, PID does not present an advantage here as a **feature selection method**. These results should be **clearly articulated** in the results section and conclusions should be drawn aptly.

We appreciate this comment as it allows us to clarify an essential point. We fully agree that PID should not be considered a feature selection method, nor was it presented as such in our manuscript. Our primary objective was not feature selection or classification performance optimization but rather to uncover higher-order informational structures within previously identified gene communities. The classification performance comparisons provided were solely intended to evaluate whether the informational structures revealed by PID retained discriminative power relative to the entire communities, despite significant reduction in the

number of genes. We added the following sentence to the "Experimental procedure" section to clarify our purpose:

> "We emphasize that PID is not intended as a feature selection method; our focus was instead on revealing higher-order interactions that might uncover hidden biological insights. Classification performance was assessed only as a validation step to confirm the informational value of PID-derived substructures."

The authors also state that the synergy cluster is enriched for disease-relevant pathways based on GSEA. GSEA typically involves ranking all genes by the magnitude of differential expression whereas the authors appear to be comparing predefined gene subsets identified via PID. This suggests that the approach the authors might be using is **over-representation analysis (ORA)** for identifying functional enrichment. The enrichment method used should be **clearly defined.**

We thank the reviewer for this insightful comment and clarification. Indeed, we confirm that the enrichment analyses reported in our manuscript were conducted using Over-Representation Analysis (ORA), which is appropriate for predefined gene sets identified through PID clustering, rather than traditional Gene Set Enrichment Analysis (GSEA), which relies on ranking genes by differential expression scores. To clarify this further detail, we entirely reviewed the "Biological tool" subsection.

Additionally, I couldn't access supplementary Table 3. If authors had used ORA for identifying functionally enriched pathways in their subsets, it would be helpful to **indicate the degree of overlap** between the identified clusters and gene signatures. Same should be done for the boot-strapped samples.

Overall, the results don't support the strengths of PID.

We apologize for the inconvenience regarding the supplementary materials and will ensure that all supplementary tables are fully accessible and clearly provided upon resubmission. Additionally, following the reviewer’s valuable suggestion, we will clearly report the degree of overlap between PID-derived clusters and known gene signatures as well as the overlaps obtained from bootstrapped samples. We added this information in the supplementary tables S1-S5, clearly labeled, to facilitate the reader's interpretation and validation of our results. For bootstrap samples we added another table in Appendix A1 section.
