# Supplementary material for: Unveiling complex patterns: An information-theoretic approach to high-order behaviors in microarray data
Source: PLoS One. 2025 Nov 13;20(11):e0336379. doi: 10.1371/journal.pone.0336379 (PMC12614557; doi:10.1371/journal.pone.0336379)
Supplement: Attachment — Submitted filename: Response to Reviewers.pdf [file pone.0336379.s012.pdf]

Response to Reviewers

PONE-D-24-38840R2
Unveiling Complex Patterns: An Information-Theoretic Approach to High-Order Behaviors in Microarray Data
PLOS ONE

Please submit your revised manuscript by Sep 26 2025 11:59PM. If you will need more time than this to complete your revisions, please reply to this message or contact the journal office at plosone@plos.org. When you're ready to submit your revision, log on to https://www.editorialmanager.com/pone/ and select the 'Submissions Needing Revision' folder to locate your manuscript file.

Please include the following items when submitting your revised manuscript:

* A rebuttal letter that responds to each point raised by the academic editor and reviewer(s). You should upload this letter as a separate file labeled 'Response to Reviewers'.
* A marked-up copy of your manuscript that highlights changes made to the original version. You should upload this as a separate file labeled 'Revised Manuscript with Track Changes'.
* An unmarked version of your revised paper without tracked changes. You should upload this as a separate file labeled 'Manuscript'.

6. Review Comments to the Author

Please use the space provided to explain your answers to the questions above. You may also include additional comments for the author, including concerns about dual publication, research ethics, or publication ethics. (Please upload your review as an attachment if it exceeds 20,000 characters)

Reviewer #1: I misunderstood the authors' meaning of biological validation. They used computer tools for validation and no biological experiments were performed.

Yes, with biological validation we intend Differential Gene Expression (DGE) analysis and Pathways Analysis.

Reviewer #2: In their comments and edits, the authors have successfully clarified the methods used for gene enrichment analysis. They also emphasized that PID is not a feature selection method, but rather a method for revealing higher order interactions that may uncover hidden biological insights. The biological test for PID identified genes is based on the analysis of differentially expressed genes (DEGs) and enriched pathways. Their DEG analysis uses an arbitrary absolute log fold change (LFC) cut-off of 1.5. The conclusions might be dependent on this arbitrary choice of LFC. Could the authors explore the results with no LFC cut-off and with an absolute LFC cut-off of 1?

We thank the reviewer for this suggestion. To address the concern, we repeated the DEG analysis using limma's TREAT function, which directly tests whether absolute log fold changes exceed a specified threshold (δ). We evaluated δ=0 (no fold-change requirement), δ=1, and δ=1.5 (the value originally reported).

As expected, relaxing the effect-size threshold increases the number of significant DEGs: at δ=0 and δ=1, all genes in the communities reached statistical significance, whereas at δ=1.5 only a small number of genes remained significant.

We therefore retained δ=1.5 in the main text for two reasons:

1. **Biological interpretability** – A minimum fold change requirement prioritizes genes with larger, more robust differences, reducing the risk of highlighting very small but statistically significant changes that may have limited biological impact.

2. **Conservativeness** – Using δ=1.5 provides a stringent criterion, ensuring that the genes reported are those most likely to represent biologically meaningful effects.

We added a sentence on the Results section:

"We adopted this logFC threshold in order to focus on genes with the largest and most biologically meaningful expression differences, providing a conservative filter that prioritizes robust disease-related signals over subtle but potentially less interpretable changes."

Additionally, the identification of a small number of unique DEGs captured within the synergy cluster may not be a reliable test for biology. I urge the authors to evaluate whether the genes identified within the synergy cluster (unique and shared) show stronger associations in this group than would be expected if the groups were random (random subjects within the community, matched in size to the patients and controls in the synergy group). Observed test statistic for each gene within the synergy group can be compared to their null

distributions to derive an empirical p-value for each gene.

As the paper stands, the evidence for biological insights gained by PID
are subjective at best.

We thank the reviewer for this constructive suggestion. Following the recommendation, we evaluated the DEG results from synergy clusters against empirical null distributions. For each community, we computed observed per-gene test statistics within the synergy group and compared them to null distributions generated from 1,000 random subject subsets matched in size and case/control composition deriving the empirical p-values for each gene.

In the HCC community 29, two disease-related DEGs were originally highlighted in synergy; under the empirical test, one gene (RBP1) did not remain significant, while the other (TCF21) retained evidence of association.

In the ASD community 50, three genes linked to disease processes emerged in synergy; two of them did not pass the empirical test, but one (PPP1R9B) remained significant compared to random subsets.

From the other two communities analysed we do not declare any information gained linked to the diseases with synergy but we have nevertheless highlighted the genes that pass the statistical test.

All these comments have been included in the manuscript in the Results section.
“To further validate these findings, we derived empirical p-values by comparing the observed gene-level statistics within synergy clusters against null distributions from 1,000 matched random subject subsets (see Supplementary Appendix S1). Among the candidate DEGs for both Synergy and MI, only TCF21 [65] remained significant under this more stringent test.”

“These two genes appear not associated with the HCC phenotype and do not remained significant comparing them against null distributions (the complete lists of genes with their corresponding empirical p values are shown in the S1 Appendix of the Supporting Information).”

“Also in this case, to further validate these findings, we derived empirical p-values by comparing the observed gene-level statistics within synergy clusters against null distributions from 1,000 matched random subject subsets (see Supplementary Appendix S2). Among the candidate DEGs, for both Synergy and MI, PPP1R9B [68], LOC286526, ZNF706 and FOXE3 remained significant under this more stringent test.”

“As far as we know, these genes don’t present a direct connection with ASD phenotype and only two genes (OR52N2 and MOGAT2) remain significant comparing them against null distributions (see S2 Appendix of the Supporting Information).”

Thus, while the empirical validation is more stringent and reduces the number of candidate genes, PID still identifies at least one robustly disease-related DEG in both HCC and ASD communities. We have added clarifying sentences in the Results to note these outcomes, and we now provide supplementary tables reporting the per-gene empirical p-values for each community where DGs are gained, so readers can transparently assess both the original and empirical results.
However, we would like to emphasize, that the principal strength of PID in this study lies not in the identification of individual DEGs from subject-as-nodes clustering, but rather in the genes-as-nodes analysis, where PID-based clustering consistently uncovered biologically meaningful enrichment patterns that were absent in mutual information clusters. Even considering the loss of some individual DEGs under the empirical test, the main conclusions of our work remain robust: PID reveals internal structures within gene communities that classical methods did not detect, and these structures align with disease-relevant biological functions.
